# Research on interpretable machine learning models for diagnosis and staging of mild cognitive impairment

**DOI:** 10.3389/fneur.2025.1708525

**Published:** 2025-11-25

**Authors:** Chongyang He, Yanyan Zhou, Yi Chen, Yang Jing

**Affiliations:** 1 Department of Radiology, Chongqing Red Cross Hospital (People’s Hospital of Jiangbei District), Chongqing, China; 2 Department of Neurology, Zunyi Medical University Affiliated Hospital, Zunyi, China; 3 Huiying Medical Technology Co., Ltd., Beijing, China

**Keywords:** mild cognitive impairment, sMRI radiomics, neuropsychological scales, plasma protein biomarkers, machine learning, SHapley Additive exPlanations

## Abstract

**Background:**

Mild cognitive impairment (MCI) is a critical prodromal stage of Alzheimer’s disease (AD), further categorized into early MCI (EMCI) and late MCI (LMCI). Early and accurate diagnosis is essential for effective prevention and intervention of AD. This study aims to develop an accessible and interpretable machine learning model to facilitate early diagnosis and subtype staging of MCI.

**Methods:**

A total of 268 participants were recruited from the ADNI, including cognitively normal individuals (CN, *n* = 132), EMCI (*n* = 95), and LMCI (*n* = 41). Participants were randomly divided into training (80%) and testing (20%) cohorts. Multimodal data encompassing whole-brain T1-WI MRI radiomics, clinical neuropsychological scales and plasma protein biomarkers were collected. Logistic regression (LR) and random forest (RF) algorithms were employed to construct six unimodal models based on above three categories of features, as well as a combined model combining all features. Diagnostic performance for the three-class classification task (CN, EMCI, LMCI) was evaluated using receiver operating characteristic (ROC) curve. Furthermore, SHapley Additive exPlanations (SHAP) were applied to quantify the contribution of individual features within the integrated model.

**Results:**

The combined model significantly outperformed unimodal models across all metrics, achieving macro_AUC = 0.92, micro_AUC = 0.91, and ACC = 0.81 in the training set, and macro_AUC = 0.87, micro_AUC = 0.87, and ACC = 0.76 in the testing set. The LR-based radiomics model ranked second. Models based solely on clinical neuropsychological scales or plasma protein biomarkers demonstrated comparatively lower classification performance. SHAP analysis highlighted neuropsychological scales (ADAS-Cog, MoCA) and radiomic features from critical brain regions (hippocampus, middle temporal gyrus, entorhinal cortex) as pivotal contributors to model efficacy.

**Conclusion:**

The integration of whole-brain structural MRI (sMRI) radiomics, neuropsychological scales, and plasma protein biomarkers significantly improves the precision of diagnosing and staging mild cognitive impairment (MCI). Radiomic characteristics derived from critical cerebral regions yield valuable pathological information that facilitates clinical interpretation. This methodology presents a promising strategy for the early identification and individualized management of MCI.

## 1 Introduction

Alzheimer’s disease (AD) is a complex, progressive neurodegenerative disorder (1). currently affecting approximately 55 million individuals worldwide, with prevalence rates anticipated to double by 2050, thereby representing a substantial challenge to global public health (2). Mild cognitive impairment (MCI) is recognized as a prodromal phase of AD (3), characterized by cognitive decline that does not yet significantly disrupt daily functional abilities (4). MCI is further categorized into early MCI (EMCI) and late MCI (LMCI) based on the extent of memory deficits (5). Longitudinal investigations indicate heterogeneous outcomes for MCI, with an annual conversion rate to AD estimated between 10 and 15% (6, 7), while a subset of patients demonstrate reversion to normative cognitive function (8). Consequently, the early and accurate diagnosis, alongside precise subtyping of MCI, is imperative for interventions aimed at delaying or preventing progression to AD, bearing significant implications for clinical practice and public health policy.

AD is pathologically defined by the accumulation of amyloid-beta (Aβ) plaques and tau neurofibrillary tangles (9), alterations that may manifest as early as the MCI stage (10). However, neuropathological confirmation is limited to postmortem examination, underscoring the urgent need for reliable *in vivo* biomarkers. Current diagnostic modalities include cerebrospinal fluid (CSF) biomarkers (e.g., Aβ42, total tau, phosphorylated tau) and positron emission tomography (PET) imaging (e.g., Aβ-PET, tau-PET, FDG-PET) (11, 12). Despite their diagnostic utility, these methods are invasive, costly, and often poorly tolerated by patients. Consequently, the diagnosis of MCI primarily relies on clinical assessment and neuropsychological instruments, notably the Mini-Mental State Examination (MMSE) (13), Montreal Cognitive Assessment (MoCA) (14), and Alzheimer’s Disease Assessment Scale-Cognitive Subscale (ADAS-Cog) (15). While these tools provide valuable clinical information, they are inherently subjective, susceptible to diagnostic inaccuracies (16), and do not reveal the underlying biological mechanisms, thereby limiting their effectiveness for early detection and targeted therapeutic intervention.

Recent research trends reveal a growing emphasis on plasma/blood based biomarkers for the continuous spectrum diagnosis of Alzheimer’s disease (17, 18). Compared to invasive CSF sampling and expensive PET imaging, plasma assays offer minimally invasive, cost-effective, and widely accessible alternatives suitable for large-scale screening and longitudinal monitoring. Nonetheless, no plasma biomarker has yet achieved gold-standard status for MCI detection, and their clinical utility requires further validation.

Numerous neuroimaging methodologies grounded in machine learning have introduced novel strategies for the early detection of MCI (19). Among those, structural MRI (sMRI) is the most widely used due to its widespread data accessibility and cost effectiveness (20, 21). Radiomics analysis enable the extraction of subtle alterations in brain morphology through high-throughput feature selection, capturing phenomena such as atrophy in critical brain regions and ventricular enlargement (22). Multiple studies have demonstrated that sMRI-based radiomics can effectively differentiate cognitively normal (CN) individuals, MCI and those with AD (23), as well as predict the transition from MCI to AD (24). Despite these encouraging findings, research focusing on the subtyping and staging of MCI remains comparatively sparse, with the scholarly discourse predominantly emphasizing model predictive accuracy rather than an in-depth exploration of clinical interpretability. The SHapley Additive exPlanations (SHAP) framework, grounded in SHapley values from cooperative game theory, offers a robust approach for interpreting machine learning model outputs by quantifying the contribution of individual features to predictions (25, 26). This interpretability facilitates enhanced clinical decision-making by providing insights that support diagnostic and therapeutic strategies.

The early diagnosis of MCI and AD remains a significant global challenge due to the scarcity of reliable and accessible diagnostic tools during the initial stages of AD (27), with the additional complexity of accurately staging MCI subtypes. This study proposes the development of a cost-effective, minimally invasive multimodal diagnostic model that integrates structural MRI radiomics, clinical neuropsychological scales, and plasma biomarkers. Utilizing machine learning techniques to integrate and optimize high-dimensional, heterogeneous datasets, the model aims to improve diagnostic accuracy and staging precision for MCI. Additionally, SHAP analysis will identify key features influencing disease progression, thereby improving the model’s clinical utility and facilitating precision medicine approaches alongside individualized intervention strategies.

## 2 Materials and methods

### 2.1 Study population

The Alzheimer’s Disease Neuroimaging Initiative (ADNI, https://adni.loni.usc.edu/), established in 2004, is a multi-center collaborative open database. Its core objective is to systematically elucidate the associative mechanisms of biomarkers—including clinical manifestations, cognitive function, imaging features, genetics, and biochemical indicators—across the entire spectrum of Alzheimer’s disease. It aims to track the complete pathological progression of the disease, ranging from normal aging to minimal symptoms, followed by mild cognitive impairment, and ultimately to dementia. Additionally, ADNI seeks to identify biomarkers applicable for diagnosis and prognosis assessment.

All raw data used in this study, including neuropsychological scales, MRI imaging, and plasma biomarkers, were obtained from the ADNI project. Inclusion criteria: (I) Possession of complete clinical data, including demography data, neuropsychological scales (MMSE, MoCA, ADAS-Cog), and C2N Diagnostics CAP/CLIA laboratory test results of plasma proteins (included concentrations of Aβ42, Aβ40, pTau217, and non-phosphorylated (np) Tau217, as well as the ratios Aβ42/Aβ40 and pTau217/npTau217). (II) Availability of complete baseline 3 T whole brain structure MRI (3D-T1-WI) original data in DICOM format, no significant motion artifacts or image distortion. (III) Strict adherence of CN, EMCI, and LMCI groupings to the original enrollment and classification criteria of ADNI. Cognitively normal participants are diagnosed if they meet the following criteria: no memory concerns; Clinical Dementia Rating (CDR) score of 0; and MMSE scores ranging from 24 to 30. MCI (including EMCI and LMCI) Common criteria: Presence of objective memory loss; CDR score of 0.5; MMSE scores ranging from 24 to 30; no impairment in other cognitive domains; and no diagnosis of dementia. Differentiation between EMCI and LMCI: Based on scores from the Wechsler Memory Scale Logical Memory II (LM-II, maximum score = 25), stratified by years of education, EMCI: For individuals with >16 years of education: LM-II scores of 9–11; 8–15 years of education: scores of 5–9; 0–7 years of education: scores of 3–6. LMCI: For individuals with >16 years of education: LM-II scores <8; 8–15 years of education: scores <4; 0–7 years of education: scores <2. Exclusion criteria: Any significant neurologic disease other than suspected incipient Alzheimer’s disease, such as Parkinson’s disease, multi-infarct dementia, Huntington’s disease, normal pressure hydrocephalus, brain tumor, progressive supranuclear palsy, seizure disorder, subdural hematoma, multiple sclerosis, or history of significant head trauma followed by persistent neurologic defaults or known structural brain abnormalities.

**Figure 1:**
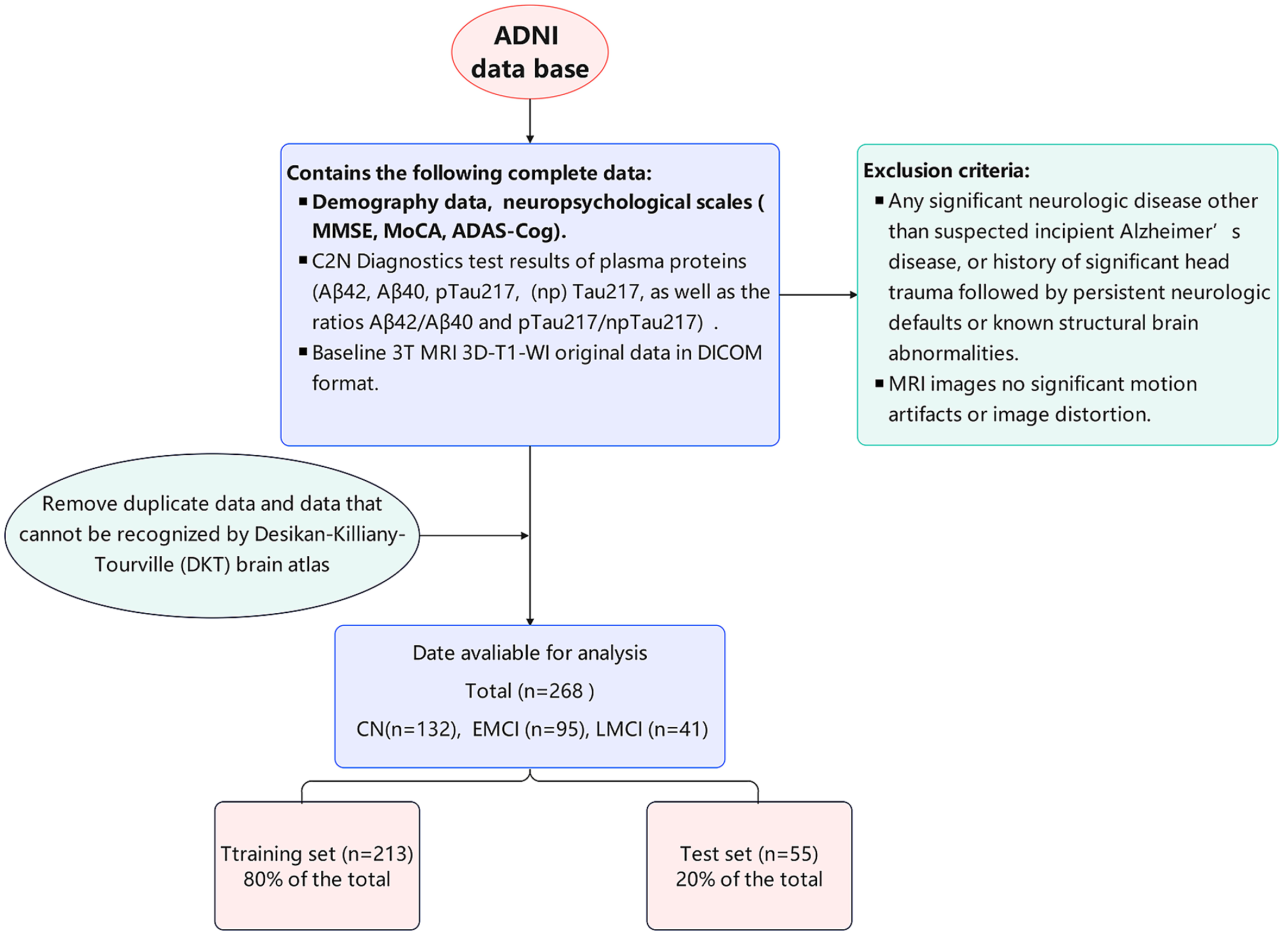
Flowchart of patient enrollment.

A total of 268 eligible patients were finally included-including 132 CN individuals-95 EMCI patients-and 41 LMCI patients. Using a stratified random sampling method (to maintain the same proportion of the three cognitive impairment groups in the training and test sets)-the patients were divided into a training set (*n* = 213-accounting for 80% of the total) and an independent test set (*n* = 55-accounting for 20% of the total), please refer to Figure 1 for this process. Ethical approval for the ADNI study was obtained from the medical ethics committees of all participating institutions, and written informed consent was obtained from all participants. This retrospective study involving human participants adhered to the ethical standards set forth by the institutional and national research committees and followed the principles outlined in the Helsinki Declaration.

### 2.2 Image acquisition and preprocessing

All brain MRI images were obtained using a 3.0 Tesla MRI scanner equipped with a 12-channel head coil. In this study, standard T1-weighted anatomical imaging was obtained by volumetric 3D magnetization-prepared rapid gradient echo (MPRAGE) or equivalent protocols with slightly different resolutions across patients. The detailed imaging protocols are provided at the ADNI website.^1^

To ensure the quality and consistency of the images for subsequent analysis-several preprocessing steps were performed. First-bias field correction was applied to the raw T1 images to eliminate the intensity inhomogeneity caused by magnetic field imperfections. This step was crucial for ensuring the accuracy of subsequent image analysis. Second-skull stripping was performed to remove non-brain tissues from the images-allowing for more accurate segmentation and analysis of brain regions. Subsequently, interpolation resampling (with a sampling rate of 1 mm × 1 mm × 1 mm) was performed on the MRI images of all patients. The preprocessed images were then segmented using the Desikan–Killiany–Tourville (DKT) brain atlas-which is a standardized template based on large-scale normal population brain structure data and can achieve automated segmentation of 95 brain regions. This segmentation process was essential for extracting radiomic features from specific brain regions related to cognitive impairment.

### 2.3 Image segmentation and feature extraction

**Figure 2:**
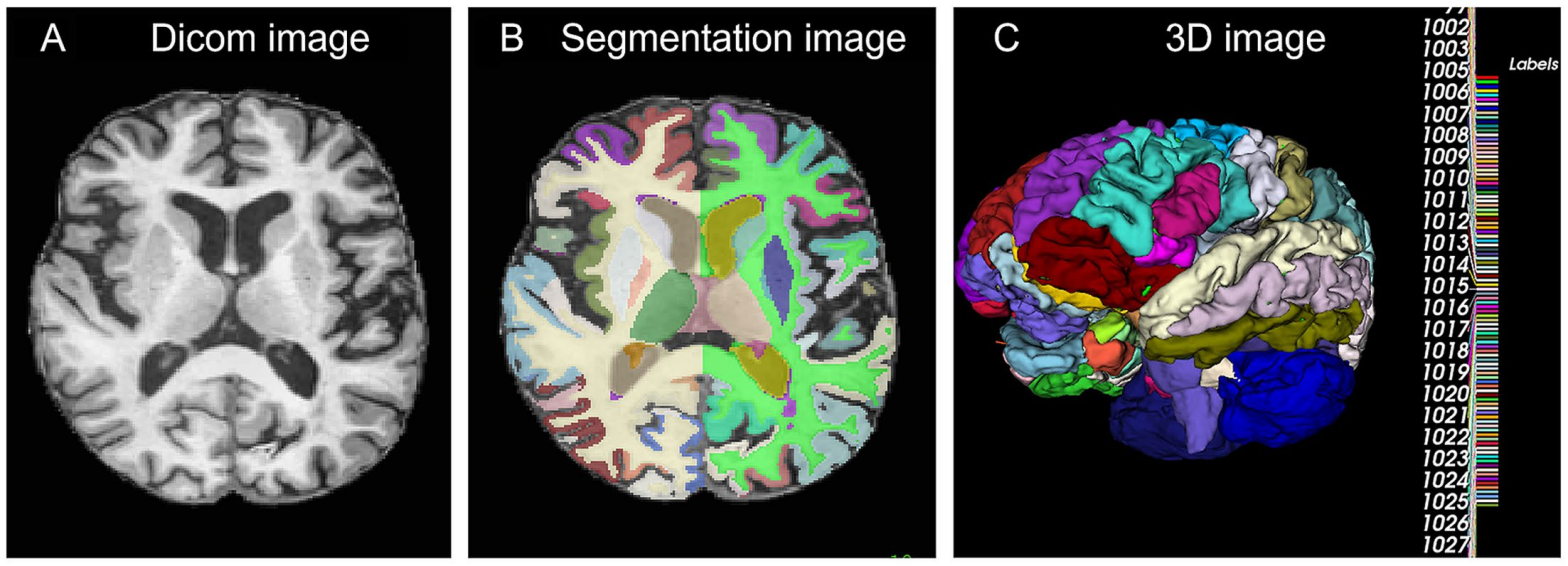
Schematic diagram of patient segmentation. **(A)** Shows the cross-sectional screenshot of the original T1 image. **(B)** Shows the Fastsurfer segmentation screenshot of the original image. **(C)** Shows the 3D rendered image after segmentation.

Images of all patients were subjected to automatic brain region segmentation using the asegdkt module of FastSurfer^2^ (28)-which can generate anatomical segmentation results and cortical parcellation of 95 brain regions based on the DKT atlas (Figure 2). The segmentation results obtained were subsequently utilized for radiomic feature extraction. By selecting all brain regions for feature extraction, this method facilitates a comprehensive characterization of the structural and morphological attributes of each region. Cognitive impairment—particularly the progressive transition from EMCI to LMCI—is frequently associated with coordinated microstructural alterations spanning multiple brain regions, rather than isolated changes confined to a single area. This comprehensive approach mitigates the risk of overlooking critical biomarkers that may arise from pre-selecting specific brain regions, thereby providing a more complete imaging feature set for the development of classification models. Consequently, this strategy enhances the model’s capacity to discriminate among the three cognitive states: CN, EMCI, and LMCI.

Radiomics features were extracted from the 95 automatically segmented brain regions using the open-source PyRadiomics package (version 3.0.1; https://pyradiomics.readthedocs.io/)-a widely validated tool for standardized radiomic feature extraction in medical imaging research. A total of three categories of radiomics features were extracted from each segmented brain region, covering comprehensive structural and textural characteristics of the brain tissue: (I) First-order statistical features (*n* = 18): These quantify the distribution of voxel intensities within each region, including metrics such as mean, variance, skewness, kurtosis, median, minimum/maximum intensity, and interquartile range, which reflect the overall tissue density and homogeneity; (II) Shape features (*n* = 14): These describe the geometric properties of each brain region, such as volume, surface area, sphericity, aspect ratio, and compactness, capturing differences in regional anatomical morphology between CN, EMCI, and LMCI groups; (III) Texture features (*n* = 75): Derived from gray-level co-occurrence matrix (GLCM), gray-level run-length matrix (GLRLM), gray-level size zone matrix (GLSZM), and gray-level dependence matrix (GLDM), these features include contrast, correlation, energy, entropy, run-length non-uniformity, and zone size non-uniformity, which characterize spatial heterogeneity and tissue microstructural patterns. After extraction, features with missing values or zero variance across all patients were excluded upfront to ensure data quality, resulting in an initial pool of 107 radiomics features per brain region.

### 2.4 Feature selection

A three-stage sequential feature selection approach was implemented to reduce dimensionality by removing redundant or irrelevant features in training set, thereby preserving the most discriminative features for the development of a three-class classification model (CN vs. EMCI vs. LMCI). This strategy aimed to prevent overfitting and enhance the interpretability of the model. A three-step sequential feature selection strategy was employed to reduce dimensionality-eliminate redundant or irrelevant features-and retain the most discriminative features for constructing the three-classification model (CN vs. EMCI vs. LMCI)-thereby avoiding overfitting and improving model interpretability.

First-variance thresholding was applied with a threshold of 0.8 (29): Features with variance lower than this threshold (i.e., features that showed minimal variation across all patients) were removed. Second-univariate analysis was performed using one-way analysis of variance (ANOVA) for continuous features: Only features with a statistical significance of *p* < 0.05 were retained-ensuring that the selected features exhibited significant differences among the three cognitive groups. Third-multi-class least absolute shrinkage and selection operator (LASSO) regression was applied to the remaining features: This method imposes a penalty on feature coefficients-shrinking irrelevant feature coefficients to zero and selecting only those with non-zero coefficients.

### 2.5 Model construction

In the construction of classification models for cognitive impairment (CI) prediction-we employed logistic regression (LR) and random forest (RF) algorithms. Logistic regression was chosen for its simplicity and interpretability-making it a reliable choice for binary classification tasks. Random forest-on the other hand-was selected due to its robustness against overfitting and ability to handle non-linear relationships in the data.

We constructed six individual models based on three types of feature sets: radiomics features derived from MRI images-clinical features obtained from neuropsychological scales-and plasma protein features measured from blood samples. Clinical and protein features were subjected to feature selection using the random forest algorithm-and the selected features were used for model construction. Additionally, a combined model was developed by integrating the feature sets using the algorithm that demonstrated superior performance in preliminary evaluations. This approach aimed to leverage the strengths of each feature type and improve the overall predictive accuracy of the model.

### 2.6 Statistical analysis

The performance of the models was assessed utilizing multiple statistical metrics-including micro and macro AUC (area under the curve)-accuracy-sensitivity-and specificity. The micro AUC was derived by aggregating all classes collectively, whereas the macro AUC was obtained by averaging the AUC values computed for each individual class. Accuracy measured the overall correctness of the model-sensitivity indicated the model’s ability to correctly identify positive cases-and specificity reflected the model’s ability to correctly recognize negative cases. All statistical analyses were conducted using Python Version 3.9.0 with relevant packages such as scikit-learn for model development and evaluation-and pandas for data processing.

## 3 Results

### 3.1 Patient characteristics

The study cohort consisted of 268 people-including 132 with cognitive normal (CN)-95 with early mild cognitive impairment (EMCI)-and 41 with late mild cognitive impairment (LMCI). The mean age was 72 years for CN-69.60 years for EMCI-and 69.34 years for LMCI-there was significant difference in age distribution among the three groups (*p* = 0.008). The gender distribution showed that 63 (47.7%) CN-57 (60.0%) EMCI patients-and 20 (48.8%) LMCI patients were male-with no significant difference in gender proportion across the groups (*p* = 0.168). However-significant differences were observed in several cognitive assessment scores and biomarkers among the groups. For instance-the median Alzheimer’s Disease Assessment Scale-Cognitive Subscale (ADAS-Cog11) score increased from 5.16 in CN to 10.00 in LMCI (*p* < 0.001)-reflecting the progressive cognitive impairment. In terms of biomarkers-the median pTau217 level was 0.65 for CN-1.34 for EMCI-and 2.19 for LMCI (*p* = 0.017)-suggesting an increase in tau pathology as the cognitive impairment worsened (Table 1).

**Table 1:** Characteristic baseline of patients.

Variable	CN (*N* = 132)	EMCI (*N* = 95)	LMCI (*N* = 41)	*p*-value
PTGENDER (%)				0.168
Male	63 (47.7)	57 (60.0)	20 (48.8)	
Female	69 (52.3)	38 (40.0)	21 (51.2)	
Age [mean (SD)]	72.00 (5.86)	69.60 (6.75)	69.34 (7.68)	0.008
PTEDUCAT [median (IQR)]	16.00 [15.00–18.00]	16.00 [14.00–18.00]	16.00 [15.00–18.00]	0.434
MMES [median (IQR)]	29.00 [29.00–30.00]	29.00 [28.00–30.00]	28.00 [26.00–29.00]	<0.001
ADAS-cog11 [median (IQR)]	5.16 [3.25–7.00]	6.33 [5.00–9.00]	10.00 [8.33–11.67]	<0.001
ADAS-cog13 [median (IQR)]	8.33 [5.00–10.42]	10.67 [7.84–14.16]	17.00 [12.67–20.67]	<0.001
Visuospatial_executive_function [median (IQR)]	5.00 [4.00–5.00]	4.00 [4.00–5.00]	4.00 [3.00–5.00]	0.001
Naming_score [median (IQR)]	3.00 [3.00–3.00]	3.00 [3.00–3.00]	3.00 [3.00–3.00]	0.049
Attention_score [median (IQR)]	6.00 [5.00–6.00]	6.00 [5.00–6.00]	5.00 [4.00–6.00]	<0.001
Language_score (median [IQR])	3.00 [2.00–3.00]	3.00 [2.00–3.00]	2.00 [2.00–3.00]	0.007
Abstract_thinking_score [median (IQR)]	2.00 [2.00–2.00]	2.00 [2.00–2.00]	2.00 [1.00–2.00]	0.085
Delayed_memory_score [median (IQR)]	2.00 [1.00–4.00]	1.00 [0.00–3.00]	0.00 [0.00–2.00]	<0.001
Orientation_score [median (IQR)]	6.00 [6.00–6.00]	6.00 [6.00–6.00]	6.00 [5.00–6.00]	<0.001
MOCA.total [median (IQR)]	26.00 [24.00–28.00]	24.00 [22.00–26.00]	22.00 [20.00–24.00]	<0.001
MOCA.adjusted.total [median (IQR)]	26.00 [24.00–28.00]	24.00 [22.00–26.00]	22.00 [20.00–24.00]	<0.001
Abeta40 [median (IQR)]	395.33 [357.01–442.00]	385.94 [347.52–424.42]	391.98 [355.27–433.79]	0.404
Abeta42 [median (IQR)]	36.64 [31.93–41.91]	37.58 [33.18–41.82]	34.16 [31.30–38.56]	0.217
Abeta_ratio [median (IQR)]	0.09 [0.09–0.10]	0.10 [0.09–0.10]	0.09 [0.09–0.10]	0.013
pTau217 [median (IQR)]	0.65 [0.65–2.16]	1.34 [0.65–2.90]	2.19 [0.65–3.76]	0.017
npTau217 [median (IQR)]	49.44 [40.84–59.63]	46.34 [40.40–54.86]	49.65 [42.66–56.67]	0.308
pTau217_ratio [median (IQR)]	2.21 [1.44–4.17]	2.37 [1.54–5.74]	3.94 [1.73–8.61]	0.013

ADAS-Cog, Alzheimer’s Disease Assessment Scale-Cognitive (delayed word recall and number cancellation will be conducted in addition to the 11 standard ADAS-Cog Items).

Abeta_ratio, Aβ42/Aβ40; pTau217_ratio, pTau217/npTau217.

MMSE, Mini Mental State Examination.

MoCA, Montreal Cognitive Assessment (educational bias adjustment: add 1 point to the total score for individuals with ≤12 years of education).

### 3.2 The results of feature selection

**Figure 3:**
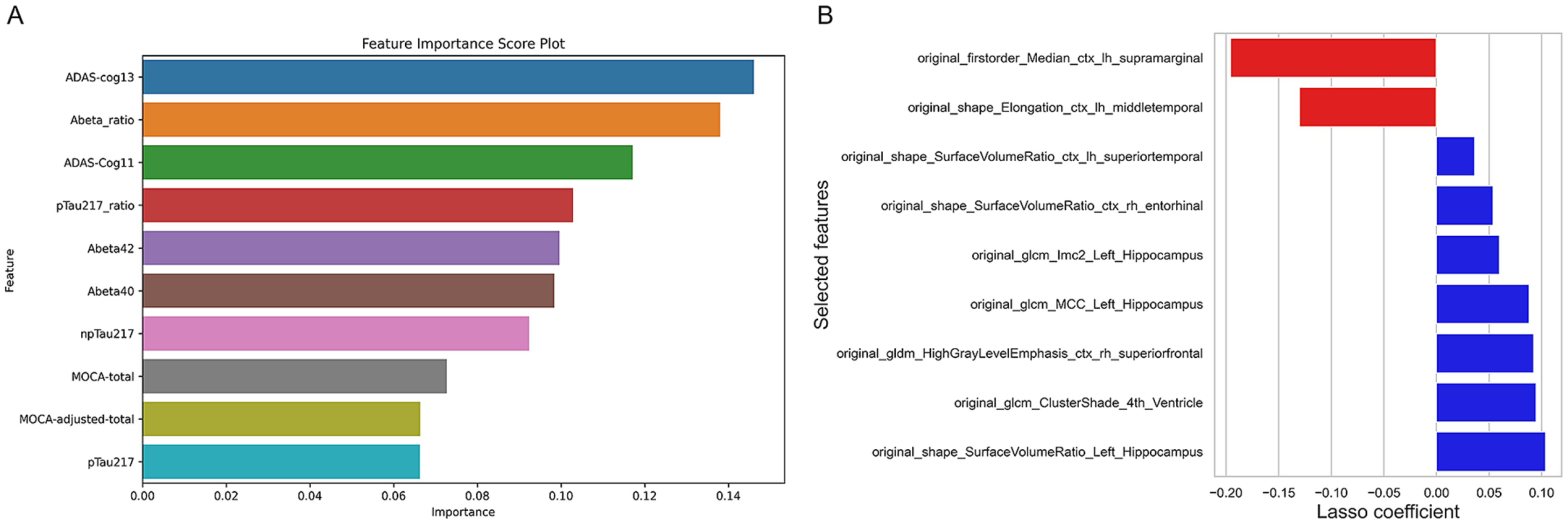
Results of feature selection. **(A)** Results chart of clinical and protein feature selection. **(B)** Results chart of radiomics feature selection.

Clinical and protein features were selected using the random forest algorithm. The selection process identified several key features associated with cognitive impairment progression. For instance-ADAS-Cog11-ADAS-cog13-MOCA-total and MOCA-adjusted-total were retained as significant clinical features. Among the protein features-Abeta42-pTau217-and their ratios were selected-indicating their potential role in distinguishing different stages of cognitive impairment (Figure 3A).

Radiomic features were extracted from MRI images-and after feature selection-10 radiomic features were retained (Figure 3B). These features reflect the morphological and textural changes in specific brain regions-such as the entorhinal cortex and hippocampus-which are critical for cognitive function. The coefficients of these features suggest their contribution to the classification model-with higher coefficients indicating a stronger association with the cognitive impairment status.

### 3.3 Model performance

**Figure 4:**
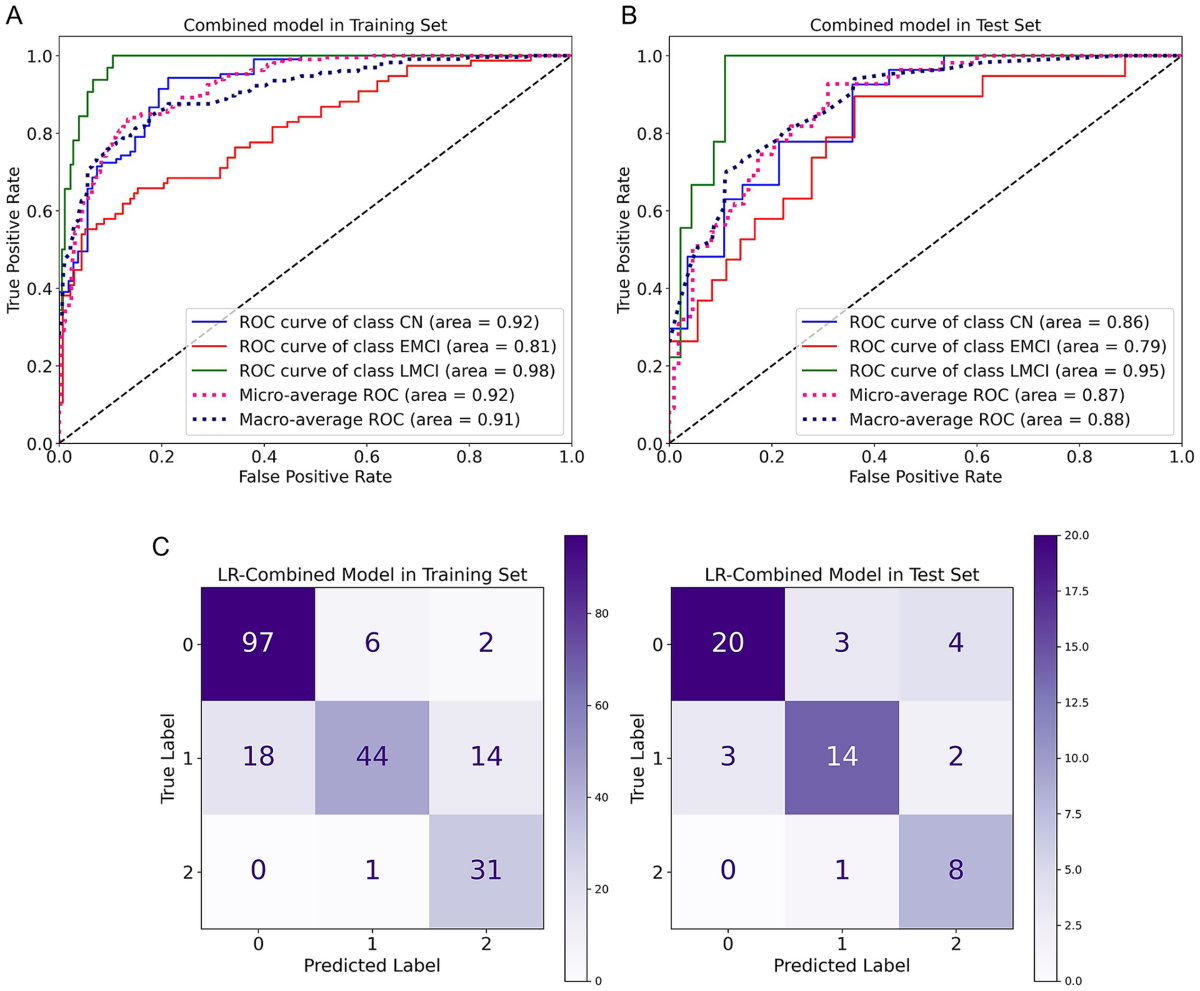
Evaluation results of the combined model. **(A,B)** ROC curves of the combined model in the training set and test set. **(C)** Confusion matrix of the combined model.

A total of seven three-classification models (CN vs. EMCI vs. LMCI) were constructed based on two algorithms [logistic regression (LR) and random forest (RF)] and three feature sets [radiomics (RS)-clinical-protein]-including six unimodal models and one combined model (integrating RS-clinical-and protein features). For LR models (Table 2)-the combined model exhibited the highest discriminative performance: in the training set-it achieved a micro-AUC of 0.92 (95% CI: 0.90–0.94) and macro-AUC of 0.91 (95% CI: 0.88–0.93) (Figures 4A,B)-with micro-sensitivity (SEN)-micro-specificity (SPE)-micro-precision-and micro-F1 all reaching 0.81; in the test set-it maintained a micro-AUC of 0.87 (95% CI: 0.80–0.93) and macro-AUC of 0.87 (95% CI: 0.80–0.93)-with micro-SEN-micro-SPE-micro-precision-and micro-F1 at 0.76. The RS model followed-with a test set micro-AUC of 0.84 (95% CI: 0.76–0.90)-while the clinical and protein models showed relatively lower performance (test set micro-AUC: 0.75 and 0.72-respectively). For RF models (Table 3)-the RS model performed best-with a training set micro-AUC of 0.88 (95% CI: 0.85–0.92) and test set micro-AUC of 0.80 (95% CI: 0.70–0.89); the clinical and protein models had test set micro-AUCs of 0.79 and 0.78-respectively. Notably-the LR-based combined model outperformed all unimodal models and RF-based models-confirming the synergistic value of multi-modal feature integration.

**Table 2:** Performance of different LR models.

Model	Set	macro_AUC (95% CI)	micro_AUC (95% CI)	SEN (ma/mi)	SPE (ma/mi)	ACC
Combined	Train	0.92 (0.9–0.94)	0.91 (0.88–0.93)	0.82/0.81	0.9/0.81	0.81
	Test	0.87 (0.8–0.93)	0.87 (0.8–0.93)	0.79/0.76	0.88/0.76	0.76
Clinical	Train	0.77 (0.72–0.81)	0.72 (0.67–0.76)	0.58/0.56	0.77/0.56	0.56
	Test	0.75 (0.66–0.84)	0.71 (0.63–0.79)	0.57/0.56	0.76/0.56	0.56
Protein	Train	0.79 (0.74–0.83)	0.76 (0.71–0.81)	0.58/0.58	0.77/0.58	0.58
	Test	0.72 (0.63–0.82)	0.74 (0.65–0.82)	0.59/0.53	0.75/0.53	0.53
RS	Train	0.91 (0.88–0.94)	0.89 (0.86–0.92)	0.8/0.79	0.89/0.79	0.79
	Test	0.84 (0.76–0.9)	0.83 (0.75–0.89)	0.73/0.75	0.87/0.75	0.75

ACC, accuracy; SEN, sensitivity; ma, macro; mi, micro.

**Table 3:** Performance of different RF models.

Model	Set	macro_AUC (95% CI)	micro_AUC (95% CI)	SEN (ma/mi)	SPE (ma/mi)	ACC
Clinical	Train	0.82 (0.78–0.86)	0.78 (0.73–0.83)	0.56/0.61	0.77/0.61	0.61
	Test	0.79 (0.69–0.86)	0.78 (0.67–0.86)	0.55/0.58	0.76/0.58	0.58
Protein	Train	0.8 (0.76–0.84)	0.76 (0.7–0.81)	0.54/0.58	0.75/0.58	0.58
	Test	0.78 (0.69–0.86)	0.76 (0.67–0.84)	0.58/0.6	0.8/0.6	0.6
RS	Train	0.88 (0.85–0.92)	0.86 (0.82–0.91)	0.67/0.76	0.86/0.76	0.76
	Test	0.8 (0.7–0.89)	0.78 (0.66–0.88)	0.73/0.75	0.86/0.75	0.75

ACC, accuracy; SEN, sensitivity; ma, macro; mi, micro.

Confusion matrices further validated model performance. For the LR-Combined Model (Figure 4C)-in the training set-it accurately classified 97 samples of the true label 0–44 of true label 1-and 31 of true label 2 [true positives (TP)]-with only a few misclassifications between different labels (e.g., 6 samples of true label 0 misclassified as label 1). In the test set-TP counts were 20-14-and 8 for true labels 0-1-and 2 respectively-and misclassification rates were relatively low. In contrast-unimodal models (such as LR-Clinical-LR-Protein) exhibited higher misclassification rates. For instance-in the LR-Protein Model’s test set-12 samples of true label 1 were misclassified as label 0-and 5 as label 2. As for RF models-though the RF-RS Model had a certain classification performance-with 14 samples of true label 1 correctly classified in the test set-it still fell short of the LR-Combined Model. These results suggested that the LR -Combined Model not only achieved high overall performance but also possessed excellent category -specific classification ability-reducing misclassifications among different categories.

### 3.4 Explainable analysis

SHapley Additive exPlanations (SHAP) was used to interpret the decision-making process of the LR combined model-quantifying the contribution of each feature to the three-classification prediction and visualizing results via SHAP bar plots (feature weight ranking) and SHAP beeswarm plots (feature value-impact relationship).

**Figure 5:**
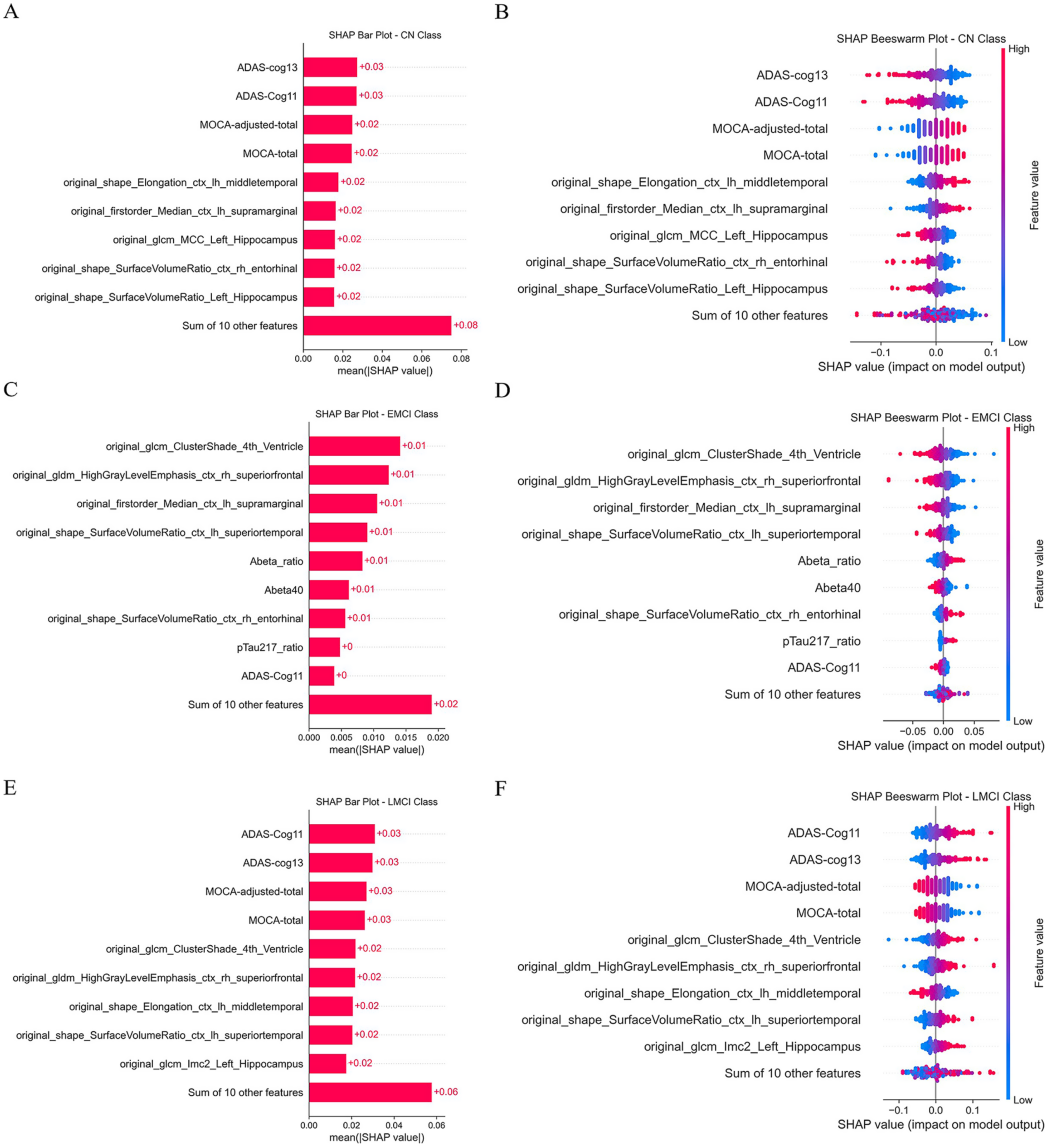
SHAP analysis plot results. **(A,C,E)** Are SHAP analysis bar plots for the CN-EMCI and CN-LMCI categories respectively. **(B,D,F)** Are SHAP analysis swarm plots for the CN-EMCI and CN-LMCI categories respectively.

For CN classification (Figures 5A,B)-the top contributing features included clinical indicators (ADAS-Cog11-ADAS-Cog13-MOCA-adjusted-total-MOCA-total) and radiomics features (e.g., original_shape_SurfaceVolumeRatio_Left_Hippocampus-original_glcm_MCC_Left_Hippocampus). Among these-lower ADAS-Cog11/13 scores (indicating better cognitive function) and higher MOCA scores had positive SHAP values (promoting CN prediction)-while higher SurfaceVolumeRatio of the left hippocampus (reflecting intact hippocampal structure) also contributed to CN classification. For EMCI classification (Figures 5C,D)-the key discriminative features exhibited balanced contributions with mean SHAP values = 0.01-including radiomics features and protein markers (Abeta_ratio-Abeta40-pTau217_ratio). The SHAP beeswarm plot further revealed that intermediate values of these features correlated with SHAP values clustered around 0—this “intermediate feature value” pattern effectively distinguished EMCI from CN (lower feature values) and LMCI (higher feature values). For LMCI classification (Figures 5E,F)-higher ADAS-Cog11/13 scores (indicating severe cognitive decline) and radiomics features such as original_glcm_ClusterShade_4th_Ventricle and original_gldm_HighGrayLevelEmphasis_ctx_rh_superiorfrontal had positive SHAP values (promoting LMCI prediction)-while lower MOCA scores further supported LMCI classification. Collectively-SHAP analysis revealed that neuropsychological scales (ADAS-Cog-MOCA) and radiomics features from key brain regions (hippocampus-middle temporal gyrus-entorhinal cortex) were the core drivers of the model-enhancing the transparency and clinical interpretability of the three-classification prediction.

## 4 Discussion

This research employed the ADNI database to develop and validate an interpretable multimodal biomarker machine learning model by integrating structural MRI radiomics features, clinical variables, and plasma protein markers. The resulting model offers a robust framework for the diagnosis and staging of mild cognitive impairment (MCI) and its subtypes, including early MCI (EMCI) and late MCI (LMCI). Findings indicate that the combined model outperforms models based on any single modality in terms of diagnostic accuracy and staging capability. Furthermore, the application of the SHAP interpretability technique identified critical pathological features influencing the model’s predictions, thereby substantially enhancing its clinical relevance and providing a scientific foundation for precision medicine and personalized intervention strategies.

### 4.1 Clinical model

In this study, the CN group exhibited a higher mean age (72 years) compared to the EMCI group (69.6 years) and the LMCI group (69.34 years), which contradicts the typical pattern of cognitive decline with advancing age. A possible explanation is that the CN participants recruited through the ADNI cohort predominantly comprised individuals with strong cognitive reserve at advanced ages, such as those with higher educational attainment and healthier lifestyles. This phenomenon aligns with the concept of cognitive resilience (30). Conversely, the EMCI and LMCI groups may have included early-onset cases (under 65 years) characterized by more rapid pathological progression (31); however, the relatively small sample size may have exaggerated the observed age differences. Consequently, age was treated solely as a demographic variable and was excluded from feature selection in the modeling process. Among neuropsychological scales, we selected MMSE, ADAS-Cog, and MoCA, which provide critical information on patients’ cognitive and functional status (32). Feature selection incorporated ADAS-Cog (11-item and 13-item versions) and MoCA (total score and education-adjusted total score) into the modeling, playing an important role in prediction. A study comparing the diagnostic accuracy of these three cognitive scales for AD and MCI showed that ADAS-Cog had the best Youden’s index and sensitivity for detecting AD and MCI, followed by MoCA (33). Notably, MoCA demonstrated significantly higher sensitivity than MMSE for both Alzheimer’s disease (0.912 vs. 0.874) and MCI (0.845 vs. 0.757), highlighting its potential as a more sensitive screening tool. In this study, ADAS-Cog 11/13 and MoCA scores (adjusted or unadjusted) showed significant differences among groups (*p* < 0.001), reflecting the progressive cognitive decline from CN to EMCI and then LMCI. SHAP analysis indicated that ADAS-Cog features contributed more than MoCA, consistent with previous research. However, the overall accuracy of the clinical model was relatively modest, with training set accuracy (ACC) = 0.56 (logistic regression, LR)/0.61 (random forest, RF) and test set ACC = 0.56 (LR)/0.58 (RF). This phenomenon may be attributed to the reliance of cognitive assessment scales on subjective patient self-reports and the evaluators’ expertise, rendering them vulnerable to confounding influences such as emotional state and fatigue.

### 4.2 Plasma protein model

Recent studies have shown that plasma pTau217 can predict brain amyloid levels in early AD (34, 35). In this study, feature selection for the plasma protein model included concentrations of Aβ42, Aβ40, pTau217, and non-phosphorylated (np) Tau217, as well as the ratios Aβ42/Aβ40 and pTau217/npTau217, providing more comprehensive data than other models. Results showed median pTau217 levels of 0.65 in the CN group, 1.34 in EMCI, and 2.19 in LMCI (*p* = 0.017); median pTau217_ratio values were 2.21 (CN), 2.37 (EMCI), and 3.94 (LMCI) (*p* = 0.013), indicating a continuous increase in plasma tau protein levels with cognitive decline, consistent with other studies and disease progression. The plasma protein model demonstrated relatively stable classification performance in both training and test sets, but with slightly lower accuracy and sensitivity, suggesting that pathological changes in plasma proteins are less distinct between MCI subtypes than in AD, aligning with the pathological progression of AD. The relatively weak performance of both clinical and protein models as single modalities reflects the limitations of single-dimensional data, but their low cost and ease of acquisition make them suitable as primary screening tools.

### 4.3 Radiomics model

This study employed a rigorous feature selection process, integrating variance thresholding, univariate selection, and the LASSO algorithm, to identify a set of radiomic features with clear biological significance for model construction. The final selection comprised 10 features (Figure 3B), which were predominantly localized in the entorhinal cortex, left hippocampus, and temporal lobe. These critical brain regions are closely associated with cognitive function and align well with the established pathological mechanisms of Alzheimer’s disease and mild cognitive impairment, such as early involvement of the entorhinal cortex and hippocampus (36, 37) and cognitive domain impairments linked to the temporal lobe (38). The radiomics model built on these 10 features showed the best performance among single modalities: training set macro AUC = 0.91 (LR)/0.88 (RF), micro AUC = 0.89 (LR)/0.86 (RF); test set macro AUC = 0.84 (LR)/0.80 (RF), micro AUC = 0.83 (LR)/0.78 (RF). Reduced surface volume ratios in the entorhinal cortex, temporal lobe, and left hippocampus directly reflect progressive atrophy in these regions during disease progression, serving as “macroscopic indicators” for subtype differentiation. Texture features (e.g., GLM_MCC, GLM_Imc2, GLM_Cluster Shade) capture microstructural heterogeneity within brain regions; changes in texture features of the fourth ventricle, temporal lobe, and hippocampus may reflect microscopic pathology such as neuronal loss and gliosis (39), serving as “microscopic indicators” for early detection of potential lesions. The radiomics model’s performance approaches that of the combined model, strongly supporting structural MRI as a core biomarker for cognitive impairment.

### 4.4 Combined model

The core innovation of this study lies in integrating three different data dimensions: sMRI radiomics (RS), which directly reflects subtle brain structural damage (e.g., hippocampal atrophy, ventricular enlargement); clinical cognitive scales (Clinical), which quantify cognitive decline (e.g., memory, executive function); and plasma proteins (Protein), which capture molecular pathology (e.g., tau tangles). The combined model significantly outperformed single-modality models across all metrics (AUC, sensitivity, specificity, accuracy): training set macro AUC = 0.92 (vs. RS 0.91, Protein 0.79, Clinical 0.77), micro AUC = 0.91 (vs. RS 0.89, Protein 0.76, Clinical 0.72), ACC = 0.81 (vs. RS 0.79, Protein 0.58, Clinical 0.56); test set macro AUC = 0.87 (vs. RS 0.84, Protein 0.72, Clinical 0.75), micro AUC = 0.87 (vs. RS 0.83, Protein 0.74, Clinical 0.71), ACC = 0.76 (vs. RS 0.75, Protein 0.53, Clinical 0.56). This strongly supports that MCI is a multidimensional disease involving “pathology-cognition-clinical” interactions (40). Currently, the most commonly used method for diagnosing mild cognitive impairment (MCI) in clinical practice remains neuropsychological scales. Research has shown that for detecting MCI, the sensitivities of ADAS-cog, MoCA, and MMSE range from 0.757 to 0.869, with specificities between 0.721 and 0.835 (33). In this study, the combined model achieved a sensitivity of 0.82 and a specificity of 0.9 in the training set, indicating that its sensitivity is significantly higher than that of conventional clinical methods. Moreover, since ADNI collaborates with the National Institute on Aging, the baseline grouping of enrolled participants strictly follows clinical diagnostic criteria, further demonstrating the practical value of the combined model. Multimodal data fusion more effectively captures the complex and complementary information at different MCI stages, overcoming the limitations of single data sources and achieving higher accuracy in distinguishing MCI subtypes. Moreover, compared to invasive cerebrospinal fluid tests and expensive PET scans, the features selected in this model are more accessible, warranting broad application.

### 4.5 SHAP analysis

This study conducted a comprehensive SHAP interpretability analysis, employing bar plots and beeswarm plots to clarify the predictive mechanisms of the combined model. The findings indicate that the classification of CN individuals predominantly depends on cognitive assessments, with ADAS-Cog and MoCA emerging as principal features, underscoring the significance of “cognitive reserve” in preserving normal cognitive function. In contrast, the classification of MCI, including EMCI and LMCI, relies more substantially on radiomic biomarkers—such as ventricular texture and hippocampal morphology—and plasma biomarkers, including Aβ40, Aβ ratio, and pTau217 ratio. These biomarkers reflect the influence of “brain structural damage” and “pathological protein deposition” in cognitive deterioration. For EMCI classification, discriminative features exhibited relatively balanced contributions, likely attributable to EMCI’s intermediate status between CN and LMCI, characterized by subtler distinguishing features that complicate differentiation. In LMCI classification, the prominence of the ADAS-Cog cognitive test increased, indicating more marked cognitive decline in advanced MCI stages and enhanced sensitivity of the assessment. This stratified feature attribution offers data-driven support for precise Alzheimer’s disease subtyping, facilitating early diagnosis and targeted intervention strategies.

### 4.6 Limitations and future directions

Although this study has yielded significant findings, certain limitations persist, notably the relatively small sample size, class imbalance and the reliance on data from a single center. Future research should prioritize multicenter studies with larger sample sizes to facilitate external validation. The present model integrates clinical data, plasma protein markers, and structural MRI features, rendering it appropriate for broad screening and preliminary diagnosis. Subsequent investigations may benefit from incorporating additional biomarkers to provide a more comprehensive representation of the underlying pathology. Moreover, the inclusion of longitudinal follow-up data to assess the model’s ability to predict the risk of conversion from EMCI or LMCI to Alzheimer’s disease dementia would further augment its clinical utility.

## 5 Conclusion

The multimodal fusion and interpretable machine learning framework developed in this study for the diagnosis and staging of MCI exhibited superior classification performance, utilizing radiomics as the primary feature source complemented by clinical scales and plasma protein biomarkers. A principal advantage of this approach is its reliance on accessible, cost-effective blood assays and widely available T1-weighted structural MRI, thereby substantially reducing implementation barriers and expenses. This renders the model well-suited for large-scale population screening, community-based follow-up, and therapeutic evaluation. Furthermore, integration with SHAP interpretability analysis elucidates the contribution of critical features underlying MCI onset and progression, offering clinicians transparent and reliable decision support. Future research will prioritize extensive multicenter validation with larger cohorts, expansion of multimodal data inputs, and seamless incorporation into clinical workflows to facilitate broad adoption in precise MCI screening, staging, and personalized intervention strategies.

## Data Availability

Publicly available datasets were analyzed in this study. This data can be found here: Alzheimer’s Disease Neuroimaging Initiative (ADNI, https://adni.loni.usc.edu/).

## Footnotes

1
https://adni.loni.usc.edu/data-samples/adni-data/neuroimaging/mri/mri-scanner-protocols/

2
https://github.com/Deep-MI/FastSurfer

## References

[1] Khojaste-Sarakhsi M Haghighi SS Ghomi SMTF Marchiori E. Deep learning for Alzheimer’s disease diagnosis: a survey. Artif Intell Med. (2022) 130:102332. doi: 10.1016/j.artmed.2022.102332 35809971

[2] GBD 2019 Dementia Forecasting Collaborators. Estimation of the global prevalence of dementia in 2019 and forecasted prevalence in 2050: an analysis for the Global Burden of Disease Study 2019. Lancet Public Health. (2022) 7:e105–25. doi: 10.1016/S2468-2667(21)00249-8 34998485 PMC8810394

[3] Anderson ND. State of the science on mild cognitive impairment (MCI). CNS Spectr. (2019) 24:78–87. doi: 10.1017/S1092852918001347 30651152

[4] Petersen RC Lopez O Armstrong MJ Getchius TSD Ganguli M Gloss D . Practice guideline update summary: Mild cognitive impairment [RETIRED]: Report of the Guideline Development, Dissemination, and Implementation Subcommittee of the American Academy of Neurology. Neurology. (2018) 90:126–35. doi: 10.1212/WNL.0000000000004826 29282327 PMC5772157

[5] Aisen PS Petersen RC Donohue MC Gamst A Raman R Thomas RG . Clinical core of the Alzheimer’s disease neuroimaging initiative: progress and plans. Alzheimers Dement. (2010) 6:239–46. doi: 10.1016/j.jalz.2010.03.006, PMID: 20451872 PMC2867843

[6] Jacobs HIL Hopkins DA Mayrhofer HC Bruner E van Leeuwen FW Raaijmakers W . The cerebellum in Alzheimer’s disease: evaluating its role in cognitive decline. Brain. (2018) 141:37–47. doi: 10.1093/brain/awx194 29053771

[7] Lee MW Kim HW Choe YS Yang HS Lee J Lee H . A multimodal machine learning model for predicting dementia conversion in Alzheimer’s disease. Sci Rep. (2024) 14:12276. doi: 10.1038/s41598-024-60134-2, PMID: 38806509 PMC11133319

[8] Yu HH Tan L Jiao MJ Lv YJ Zhang XH Tan CC . Dissecting the clinical and pathological prognosis of MCI patients who reverted to normal cognition: a longitudinal study. BMC Med. (2025) 23:260. doi: 10.1186/s12916-025-04092-0, PMID: 40325426 PMC12054060

[9] Graff-Radford J Yong KXX Apostolova LG Bouwman FH Carrillo M Dickerson BC . New insights into atypical Alzheimer’s disease in the era of biomarkers. Lancet Neurol. (2021) 20:222–34. doi: 10.1016/S1474-4422(20)30440-3, PMID: 33609479 PMC8056394

[10] Abner EL Kryscio RJ Schmitt FA Fardo DW Moga DC Ighodaro ET . Outcomes after diagnosis of mild cognitive impairment in a large autopsy series. Ann Neurol. (2017) 81:549–59. doi: 10.1002/ana.24903, PMID: 28224671 PMC5401633

[11] Jack CR Andrews SJ Beach TG Buracchio T Dunn B Graf A . Revised criteria for the diagnosis and staging of Alzheimer’s disease. Nat Med. (2024) 30:2121–4. doi: 10.1038/s41591-024-02988-7, PMID: 38942991 PMC11630478

[12] Jack CR Bennett DA Blennow K Carrillo MC Dunn B Haeberlein SB . NIA-AA research framework: toward a biological definition of Alzheimer’s disease. Alzheimers Dement. (2018) 14:535–62. doi: 10.1016/j.jalz.2018.02.018, PMID: 29653606 PMC5958625

[13] Folstein MF Folstein SE McHugh PR. “Mini-mental state”. A practical method for grading the cognitive state of patients for the clinician. J Psychiatr Res. (1975) 12:189–98. doi: 10.1016/0022-3956(75)90026-6 1202204

[14] Nasreddine ZS Phillips NA Bédirian V Charbonneau S Whitehead V Collin I . The Montreal Cognitive Assessment, MoCA: a brief screening tool for mild cognitive impairment. J Am Geriatr Soc. (2025) 53:695–9. doi: 10.1111/j.1532-5415.2005.53221.x Erratum in: *J Am Geriatr Soc*. (2019) 67:1991. doi: 10.1111/jgs.15925, PMID: 15817019

[15] Rosen WG Mohs RC Davis KL. A new rating scale for Alzheimer’s disease. Am J Psychiatry. (1984) 141:1356–64. doi: 10.1176/ajp.141.11.1356 6496779

[16] 2023Alzheimer’s Association Report. Alzheimer’s disease facts and figures. Alzheimers Dement. (2023) 19:1598–695. doi: 10.1002/alz.13016 36918389

[17] Chatterjee P Pedrini S Doecke JD Thota R Villemagne VL Doré V . Plasma Aβ42/40 ratio, p-tau181, GFAP, and NfL across the Alzheimer’s disease continuum: a cross-sectional and longitudinal study in the AIBL cohort. Alzheimers Dement. (2023) 19:1117–34. doi: 10.1002/alz.12724 36574591

[18] Yakoub Y Gonzalez-Ortiz F Ashton NJ Déry C Strikwerda-Brown C St-Onge F . Plasma p-tau217 identifies cognitively normal older adults who will develop cognitive impairment in a 10-year window. Alzheimers Dement. (2025) 21:e14537. doi: 10.1002/alz.14537, PMID: 40008832 PMC11863240

[19] Ahmadzadeh M Christie GJ Cosco TD Arab A Mansouri M Wagner KR . Neuroimaging and machine learning for studying the pathways from mild cognitive impairment to Alzheimer’s disease: a systematic review. BMC Neurol. (2023) 23:309. doi: 10.1186/s12883-023-03323-2, PMID: 37608251 PMC10463866

[20] Suh CH Shim WH Kim SJ Roh JH Lee JH Kim MJ . Development and validation of a deep learning-based automatic brain segmentation and classification algorithm for Alzheimer disease using 3D T1-weighted volumetric images. AJNR Am J Neuroradiol. (2020) 41:2227–34. doi: 10.3174/ajnr.A6848, PMID: 33154073 PMC7963227

[21] Aghdam MA Bozdag S Saeed F Alzheimer’s Disease Neuroimaging Initiative. Machine-learning models for Alzheimer’s disease diagnosis using neuroimaging data: survey, reproducibility, and generalizability evaluation. Brain Inform. (2025) 12:8. doi: 10.1186/s40708-025-00252-3, PMID: 40117001 PMC11928716

[22] Fernández-Cabello S Kronbichler M Van Dijk KRA Goodman JA Spreng RN Schmitz TW . Basal forebrain volume reliably predicts the cortical spread of Alzheimer’s degeneration. Brain. (2020) 143:993–1009. doi: 10.1093/brain/awaa012 32203580 PMC7092749

[23] Lin A Chen Y Chen Y Ye Z Luo W Chen Y . MRI radiomics combined with machine learning for diagnosing mild cognitive impairment: a focus on the cerebellar gray and white matter. Front Aging Neurosci. (2024) 16:1460293. doi: 10.3389/fnagi.2024.1460293, PMID: 39430972 PMC11489926

[24] Miao D Zhou X Wu X Chen C Tian L. Hippocampal morphological atrophy and distinct patterns of structural covariance network in Alzheimer’s disease and mild cognitive impairment. Front Psychol. (2022) 13:980954. doi: 10.3389/fpsyg.2022.980954, PMID: 36160522 PMC9505506

[25] Lundberg SM Lee SI. (2017). A unified approach to interpreting model predictions. Advances in Neural Information Processing Systems 30 (NIPS 2017). Available online at: https://proceedings.neurips.cc/paper/2017/hash/8a20a8621978632d76c43dfd28b67767-Abstract.html. (Accessed September 18, 2025)

[26] Martin SA Townend FJ Barkhof F Cole JH. Interpretable machine learning for dementia: a systematic review. Alzheimers Dement. (2023) 19:2135–49. doi: 10.1002/alz.12948, PMID: 36735865 PMC10955773

[27] Porsteinsson AP Isaacson RS Knox S Sabbagh MN Rubino I. Diagnosis of early Alzheimer’s disease: clinical practice in 2021. J Prev Alzheimers Dis. (2021) 8:371–86. doi: 10.14283/jpad.2021.23, PMID: 34101796 PMC12280795

[28] Henschel L Conjeti S Estrada S Diers K Fischl B Reuter M. FastSurfer—a fast and accurate deep learning based neuroimaging pipeline. NeuroImage. (2020) 219:117012. doi: 10.1016/j.neuroimage.2020.117012, PMID: 32526386 PMC7898243

[29] Zhang MZ Ou-Yang HQ Liu JF Jin D Wang CJ Ni M . Predicting postoperative recovery in cervical spondylotic myelopathy: construction and interpretation of T2*-weighted radiomic-based extra trees models. Eur Radiol. (2022) 32:3565–75. doi: 10.1007/s00330-021-08383-x 35024949

[30] Jia B Xu Y Zhu X. Cognitive resilience in Alzheimer’s disease: mechanism and potential clinical intervention. Ageing Res Rev. (2025) 106:102711. doi: 10.1016/j.arr.2025.102711 40021093

[31] Li D Wang Y Wang J Tang Q. Identification of key proteins in early-onset Alzheimer’s disease based on WGCNA. Front Aging Neurosci. (2024) 16:1412222. doi: 10.3389/fnagi.2024.1412222, PMID: 39444808 PMC11496171

[32] Hemmy LS Linskens EJ Silverman PC Miller MA KMC T Taylor BC . Brief cognitive tests for distinguishing clinical Alzheimer-type dementia from mild cognitive impairment or normal cognition in older adults with suspected cognitive impairment: a systematic review. Ann Intern Med. (2020) 172:678–87. doi: 10.7326/M19-3889 32340040

[33] Wang X Li F Tian J Gao Q Zhu H. Bayesian estimation for the accuracy of three neuropsychological tests in detecting Alzheimer’s disease and mild cognitive impairment: a retrospective analysis of the ADNI database. Eur J Med Res. (2023) 28:427. doi: 10.1186/s40001-023-01265-6, PMID: 37821912 PMC10568914

[34] Mattsson-Carlgren N Janelidze S Palmqvist S Cullen N Svenningsson AL Strandberg O . Longitudinal plasma p-tau217 is increased in early stages of Alzheimer’s disease. Brain. (2020) 143:3234–41. doi: 10.1093/brain/awaa286, PMID: 33068398 PMC7719022

[35] Rissman RA Langford O Raman R Donohue MC Abdel-Latif S Meyer MR . Plasma Aβ42/Aβ40 and phospho-tau217 concentration ratios increase the accuracy of amyloid PET classification in preclinical Alzheimer’s disease. Alzheimers Dement. (2024) 20:1214–24. doi: 10.1002/alz.13542, PMID: 37932961 PMC10916957

[36] Enkirch SJ Traschütz A Müller A Widmann CN Gielen GH Heneka MT . The ERICA score: an MR imaging-based visual scoring system for the assessment of entorhinal cortex atrophy in Alzheimer disease. Radiology. (2018) 288:226–333. doi: 10.1148/radiol.2018171888 29514015

[37] Jaroudi W Garami J Garrido S Hornberger M Keri S Moustafa AA. Factors underlying cognitive decline in old age and Alzheimer’s disease: the role of the hippocampus. Rev Neurosci. (2017) 28:705–14. doi: 10.1515/revneuro-2016-0086 28422707

[38] Jessen F Gür O Block W Ende G Frölich L Hammen T . A multicenter ^1^H-MRS study of the medial temporal lobe in AD and MCI. Neurology. (2009) 72:1735–40. doi: 10.1212/WNL.0b013e3181a60a20 19451528

[39] Mostafa M Disouky A Lazarov O. Therapeutic modulation of neurogenesis to improve hippocampal plasticity and cognition in aging and Alzheimer’s disease. Neurotherapeutics. (2025) 22:e00580. doi: 10.1016/j.neurot.2025.e00580, PMID: 40180804 PMC12047516

[40] Anand S Schoo C. Mild cognitive impairment In: StatPearls. Treasure Island, FL: StatPearls Publishing (2025)

